# Automatic detection of microaneurysms in optical coherence tomography images of retina using convolutional neural networks and transfer learning

**DOI:** 10.1038/s41598-022-18206-8

**Published:** 2022-08-17

**Authors:** Ramin Almasi, Abbas Vafaei, Elahe Kazeminasab, Hossein Rabbani

**Affiliations:** 1 Department of Computer Engineering, Faculty of Engineering, University of Isfahan, Isfahan, Iran; 2 Medical Image and Signal Processing Research Center, Isfahan University of Medical Sciences, Isfahan, Iran

**Keywords:** Biomedical engineering, Imaging and sensing, Image processing

## Abstract

Microaneurysms (MAs) are pathognomonic signs that help clinicians to detect diabetic retinopathy (DR) in the early stages. Automatic detection of MA in retinal images is an active area of research due to its application in screening processes for DR which is one of the main reasons of blindness amongst the working-age population. The focus of these works is on the automatic detection of MAs in en face retinal images like fundus color and Fluorescein Angiography (FA). On the other hand, detection of MAs from Optical Coherence Tomography (OCT) images has 2 main advantages: first, OCT is a non-invasive imaging technique that does not require injection, therefore is safer. Secondly, because of the proven application of OCT in detection of Age-Related Macular Degeneration, Diabetic Macular Edema, and normal cases, thanks to detecting MAs in OCT, extensive information is obtained by using this imaging technique. In this research, the concentration is on the diagnosis of MAs using deep learning in the OCT images which represent in-depth structure of retinal layers. To this end, OCT B-scans should be divided into strips and MA patterns should be searched in the resulted strips. Since we need a dataset comprising OCT image strips with suitable labels and such large labelled datasets are not yet available, we have created it. For this purpose, an exact registration method is utilized to align OCT images with FA photographs. Then, with the help of corresponding FA images, OCT image strips are created from OCT B-scans in four labels, namely MA, normal, abnormal, and vessel. Once the dataset of image strips is prepared, a stacked generalization (stacking) ensemble of four fine-tuned, pre-trained convolutional neural networks is trained to classify the strips of OCT images into the mentioned classes. FA images are used once to create OCT strips for training process and they are no longer needed for subsequent steps. Once the stacking ensemble model is obtained, it will be used to classify the OCT strips in the test process. The results demonstrate that the proposed framework classifies overall OCT image strips and OCT strips containing MAs with accuracy scores of 0.982 and 0.987, respectively.

## Introduction

Nowadays, a lot of researches have been conducted on the retinal image analysis area. One of the most important applications of these works is Diabetic retinopathy (DR) screening because this disorder is one of the leading reasons for vision loss in the working-age population, especially in developed countries. Since Microaneurysms (MAs) are early signs of DR, their detection in retinal images aids screening tasks of diagnosis DR. That is why, automatic detection of MAs from retinal images is extremely important. Although several researches have been done in automatic detection of MAs, detection of MAs is still a challenging task due to the variations in MAs appearance in retinal images^1,2^. The focus of these researches is on the en face retinal images like FA and color fundus photograph. The main steps in MA detection include (1) preprocessing (2) detection of MA candidates and (3) classification of candidates. In the first step, preprocessing tackles the problems related to contrast and non-uniform illumination of MA regions. Afterwards, the initial set for MA candidates is detected and, finally some techniques (often machine learning) are applied to eliminate false-positive cases of MA and enhance the accuracy of MA detection. Many algorithms are proposed in^1–23^, each of which brings some improvements into the above-mentioned steps. Table 1 indicates some of the recent works in details.

**Table 1:** Recent works on MA detection from fundus photographs (CPM = Competition measure, CV = cross validation, BV = Blood vessel, ROC dataset = retinopathy online challenge datas et AUC = area under curve, ROC = receiver operating characteristic, SSAE = stacked sparse auto encoder, NB = Naive Bayesian, KNN = K-nearest neighbor, SVM = support vector machine, MMMF = multi-scale and multi-orientation sum of matched filter, and LLDA = local linear discrimination analysis).

Author	Dataset	Image type	Initial candidates method	Classifier for false positive reduction	Results
Habib et al. ^1^	New dataset based on the MESSIDOR dataset	Color fundus	Gaussian matched filter	Tree Ensemble classifier	ROC score = 41.5%
Hatanaka et al. ^9^	DIARETDB1 and ROC datasets	Color fundus	DCNN (GoogLeNet)	DCNN and three-layer perceptron with 48 features	Sensitivity = 84%
Shan et al. ^16^	DIARETDB dataset	Color fundus	SSAE with two hidden layers plus a Softmax classifier	AUC = 96.2%	
				With tenfold CV	
				f-score = 91.3%	
Deepa et al. ^4^	DIARETDB1 dataset	Color fundus	CNN		Sensitivity = 97.62% Specificity = 100% Accuracy = 97.75%
Zhang et al. ^23^	IDRiD_VOC dataset	Color fundus	Deep neural network with a multilayer attention mechanism	Refining initial candidates using spatial relationships between MAs and BVs	Sensitivity = 86.8%
Eftekhari et al.^5^	ROC and E-Ophtha-MA datasets	Color fundus	Basic CNN	Thresholded probability map and final CNN	CPM = 0.461
Long et al.^13^	E-Ophtha-MA and DIARETDB1 datasets	Color fundus	BV removal, shape characteristics and connected components analysis	Classification using NB, KNN and SVM	AUC -ROC = 87% on E-Ophtha-MA
					AUC of ROC = 86% on DIARETDB1
Wu et al.^21^	ROC dataset	Color fundus	MMMF	Extracting 37 dimensional features and using modified KNN, LLDA, and SVM	Averaged number of false positives per image = 0.286

On the other hand, since OCT images depict the in-depth structure of retinal layers, to the best of our knowledge, optimistically there are few researches that pursue detection of MAs in OCT photographs. As shown in Table 2, most of the researches in OCT classification present the algorithms to classify OCT volumes or OCT B-scans into the AMD, DME, and normal classes^24–27^. AMD is a retinal disease resulting in blurred, blind spots or complete loss of vision in the center of visual field and DME is the most common cause of diabetic vision loss in different societies^28^. The method presented in^29^ classifies OCT images into normal and MA categories. For this purpose, features are extracted from the images using Bag of Features (BoF) and SURF descriptor. After that, images are classified into normal and MA classes utilizing a multilayer perceptron network.

**Table 2:** Some recent works on OCT image classification considering diabetic retinopathy (MCME = multi-scale convolutional mixture of experts, LBP = local binary patterns, BoF = bag of features, SURF = speeded up robust features, and MLP = multilayer perceptron).

Author	Dataset	Image type	Method	Results
Lemaître et al.^24^	SERI Dataset of normal, DME-cyst, DME-Exudate	SD-OCT images	LBP features, different mapping strategies and using linear and nonlinear classifiers	Sensitivity = 81.2% Specificity = 93.7%
Rasti et al.^25^	Duke university dataset and private dataset of AMD, DME, and normal	Macular OCT	MCME model containing CNN experts and gating network are fed by specific scales of the input pattern	Precision = 98.86%
				AUC-ROC = 99.85%
Shih et al.^26^	Dataset of DME, CNV, Drusen, and normal^30^	OCT images	Pre-trained VGG16	Accuracy = 99.48%
Tsuji et al.^27^	Dataset of DME, CNV, Drusen, and normal^30^	OCT images	Capsule network	Accuracy = 99.6%
Kazeminasab et al.^29^	Dataset of MA and normal OCT strips	OCT images	BoF and SURF, MLP classifier	Accuracy = 94.5%

Amid the above-mentioned researches, only^29^ has focused on the detection of MAs in OCT images. Detection of MAs from OCT images has the following advantages: first, OCT is a non-invasive imaging technique that does not require injection, therefore is safer. Second, as mentioned before, OCT images are normally used for distinguishing between DME, AMD, and normal cases. Detection of MAs from OCT images results in achieving comprehensive information from this single imaging technique. This may reduce the need for using other imaging methods. It is worth mentioning that, there is another retinal imaging technique called Optical Coherence Tomography Angiography (OCTA) which is fast and non-invasive. This method provides volumetric data with the clinical capability of specifically localizing and delineating pathology along with the ability to show both structural and blood flow information in tandem. However this method has some limitations. First, this method is relatively new and not yet very common. Second, it has a relatively small field of view and is not able to show leakage well.

For doing so, we need a dataset comprising OCT image strips with suitable labels including MA, normal, abnormal, and vessel. The abnormal category includes OCT strips that contain objects in the shape of cysts and fluid-associated abnormalities in the retina. Abnormal strips do not have the normal layer structure of a normal OCT strip. To the best of our knowledge, such large labelled datasets are not yet available for OCT images, therefore we have created it. Since MAs and vessels are hard-to-detect objects in OCT images, to create OCT strips with appropriate labels, an accurate image registration method is performed to align OCT images and FA photographs. After that, with the help of corresponding FA images, the OCT strips are created from OCT B-scans in four labels, namely MA, normal, abnormal, and vessel. Once the OCT strips are created and organized as four-class dataset, a stacking ensemble^31^ comprising four fine-tuned, pre-trained CNNs is trained to classify the strips of OCT images into the mentioned classes. After the model is obtained, it classifies the strips from the test B-scans. It should be noted that FA images are used once to create OCT strips for training process. After that, the FA images are no longer needed and the stacking ensemble model classifies the OCT strips in the test process independently. To apply our model to the new test images, test B-scans should be divided into strips and the resulted strips should be fed to the stacking ensemble model to be classified into one of the above-mentioned classes. For clinical applications, to find MAs in an OCT volume, first, the B-scans are extracted, then overlapping OCT strips are created (to find all MAs) and after that, the strips are fed to the model for testing. Once the strip label is determined as MA, it can be stated in which B-scan and which strip (known location) the MA is located.

The outline of this paper is organized as follows: “Methods” section describes the data acquisition, dataset organization, and proposed stacking ensemble method used in this study. In “Results” section, the evaluation criteria and the results are presented, and “Discussion” section concludes this study.

## Methods

**Figure 1:**
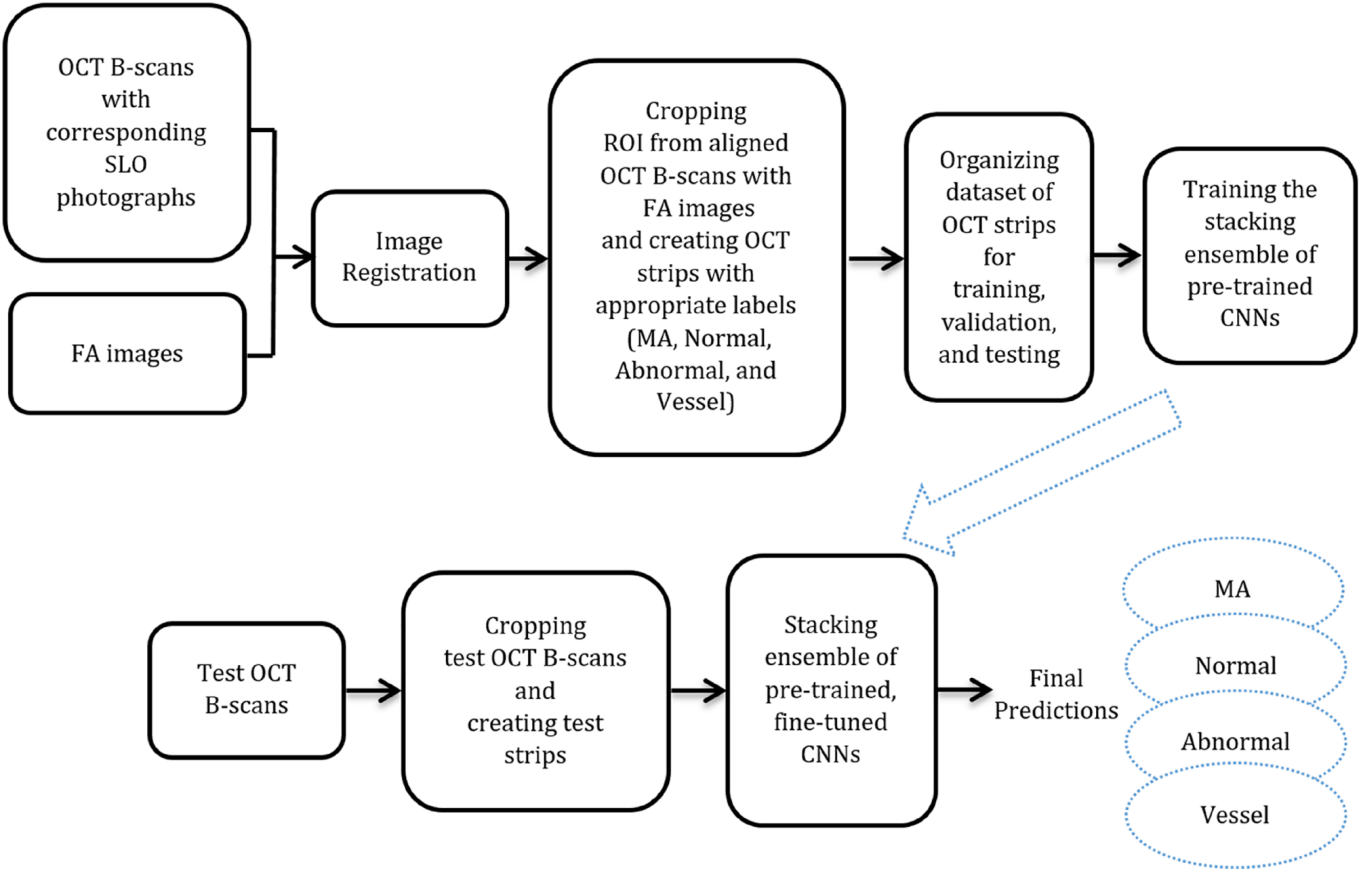
Overall block diagram of proposed method (SLO = Scanning Laser Ophthalmoscopy).

The overall block diagram of proposed method is presented in Fig. 1 and the details are explained in the next subsections.

### Image registration and OCT strips preparation

For classification purposes, we need a dataset comprising OCT image strips with suitable labels including MA, normal, abnormal, and vessel. To the best of our knowledge, such labelled datasets are not yet available for OCT images, therefore we had to create it. MAs and vessels are hard-to-detect objects in OCT images, therefore to prepare the dataset of OCT image strips with appropriate labels, a precise registration is performed to align OCT images and FA photographs. To do so, the dataset and the method proposed in^32^ is used for accurate registration of OCT and FA images using SLO photographs as intermediate images. In^32^, dataset includes 36 pairs of FA and SLO images of 21 subjects with diabetic retinopathy, where SLO image pixels are perfectly in correspondence with OCT B-scans. The FA, OCT, and SLO images are captured via Heidelberg Spectralis HRA2/OCT device. Moreover, the FA and SLO images are the same size as 768 × 768 pixels and FA images were captured with two different fields of views (30 and 55°). In this method, after preprocessing, retinal vessel segmentation is applied to extract blood vessels from the FA and SLO images. Afterwards, a global registration is used based on the Gaussian model for curved surface of retina and for this purpose, first a global rigid transformation is applied to FA vessel-map image using a feature-based method to align it with SLO vessel-map photograph and then the transformed image is globally registered again considering Gaussian model for curved surface of retina to improve the precision of the previous step. Next, a local non-rigid transformation is performed to register two images perfectly.

**Figure 2:**
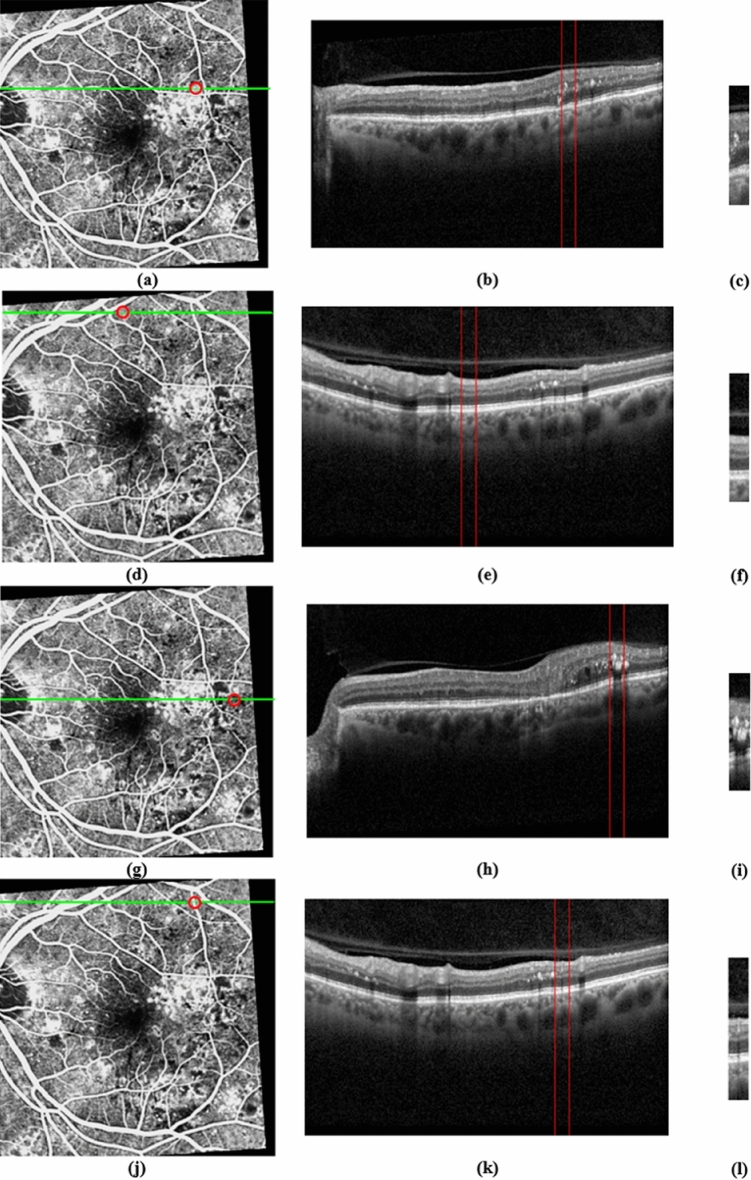
The process of creating OCT strips for MA, normal, abnormal, and vessel with the help of corresponding FA. (**a**) Red circle shows MA in FA. (**b**) B-scan Corresponds to green line in (**a**). (**c**) Cropped ROI from (**b**). (**d**–**f**) Creating strip for normal class. (**g**–**i**) Creating strip for abnormal class. (**j**–**l**) Creating strip for vessel class (in color).

After that, as shown in Fig. 2, with the help of related FAs, the OCT strips are created from OCT B-scans in four labels including MA, normal, abnormal, and vessel. The FA images are associated only once to create OCT strips for the training process. In the test process, the FA images are no longer needed. In our dataset, MA, normal, abnormal, and vessel classes comprise 87, 100, 72, and 131 strips of OCT images, respectively. In this study, the scale factor of the OCT images in the x direction equals 0.0115. So, in the OCT image, the value of x per pixel is 0.0115 mm. On the other hand, as reported in^33^, the MA has a maximum external diameter of 266 µm. Applying this to our dataset, the maximum external diameter of the MA is calculated as 23.2 pixels, approximately. Therefore, here, the width of the OCT strip is considered to be 31 pixels, which is slightly larger than 23 pixels. The images are cropped in a way that they contain only retinal layers while the other pixels are withdrawn. For this purpose, first the segmentation method presented in^34^ is used to detect the Retinal Nerve Fiber Layer (RNFL) and Retinal Pigment Epithelium (RPE) layer, afterwards the OCT B-scan is cropped to include the highest part of NFL and the lowest part of RPE. This process is demonstrated in Supplementary Fig. S1 online. That is why the images have various heights. The process of gathering the dataset of OCT strips and testing B-scans is depicted in Supplementary Figs. S2 and S3 online. It should be noted that when they are used as input for CNNs, the image will be resized to a new dimension of 150 × 150 × 3. The dataset is publicly available in https://misp.mui.ac.ir/en/four-class-dataset-oct-image-strips-png-format-%E2%80%8E-1.

### Organizing train, validation and test data

The dataset is organized into train, validation, and test folders each of which includes MA, normal, abnormal, and vessel image folders. Twenty percent of entire dataset is allocated to the test set, and is not utilized in the training process. This is referred to as Hold-out method. To validate each CNN, the Bayesian optimization tuner class of the Keras tuner^35^ is used to run the search over the search space. The search space includes the learning rate, momentum, and the number of units in the first dense layer. The number of trials and epochs in the validation process is considered to be 10 and 80, respectively. The tuned hyperparameters for each CNN are listed in Supplementary Table S1 online. Fifteen percent of the entire dataset is allocated to the validation set. Because the dataset of our work is small compared to common deep learning task datasets and this may lead to overfitting, the data augmentation technique is applied. Using this technique, some transformations including rotation, zooming, horizontal flip, re-scaling, and shift are applied to the dataset images in each single training epoch by the image data generator. This helps the model not to memorize images and as a result not to overfit.

### Stacked generalization ensemble

**Figure 3:**
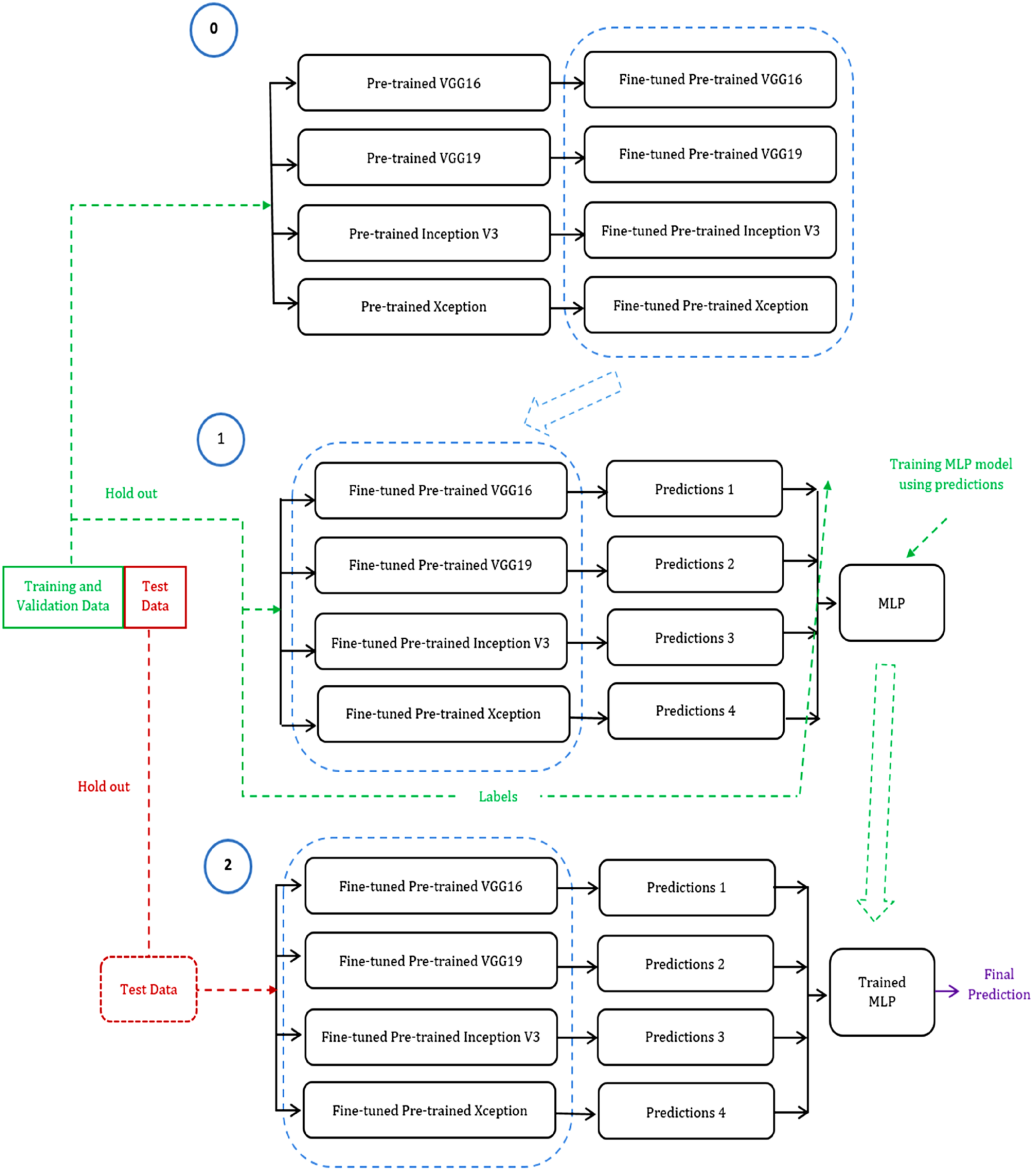
Overall structure of the stacked generalization ensemble.

The overall structure of the classifier presented in this research is shown in Fig. 3. As can be observed, the stacked generalization (stacking) ensemble of four CNNs pretrained on the ImageNet dataset is utilized. Stacking ensemble includes two levels, namely 0 and 1. The elements and training process of each level is elaborated in the next sections.

#### Level 0 of stacking ensemble

**Figure 4:**
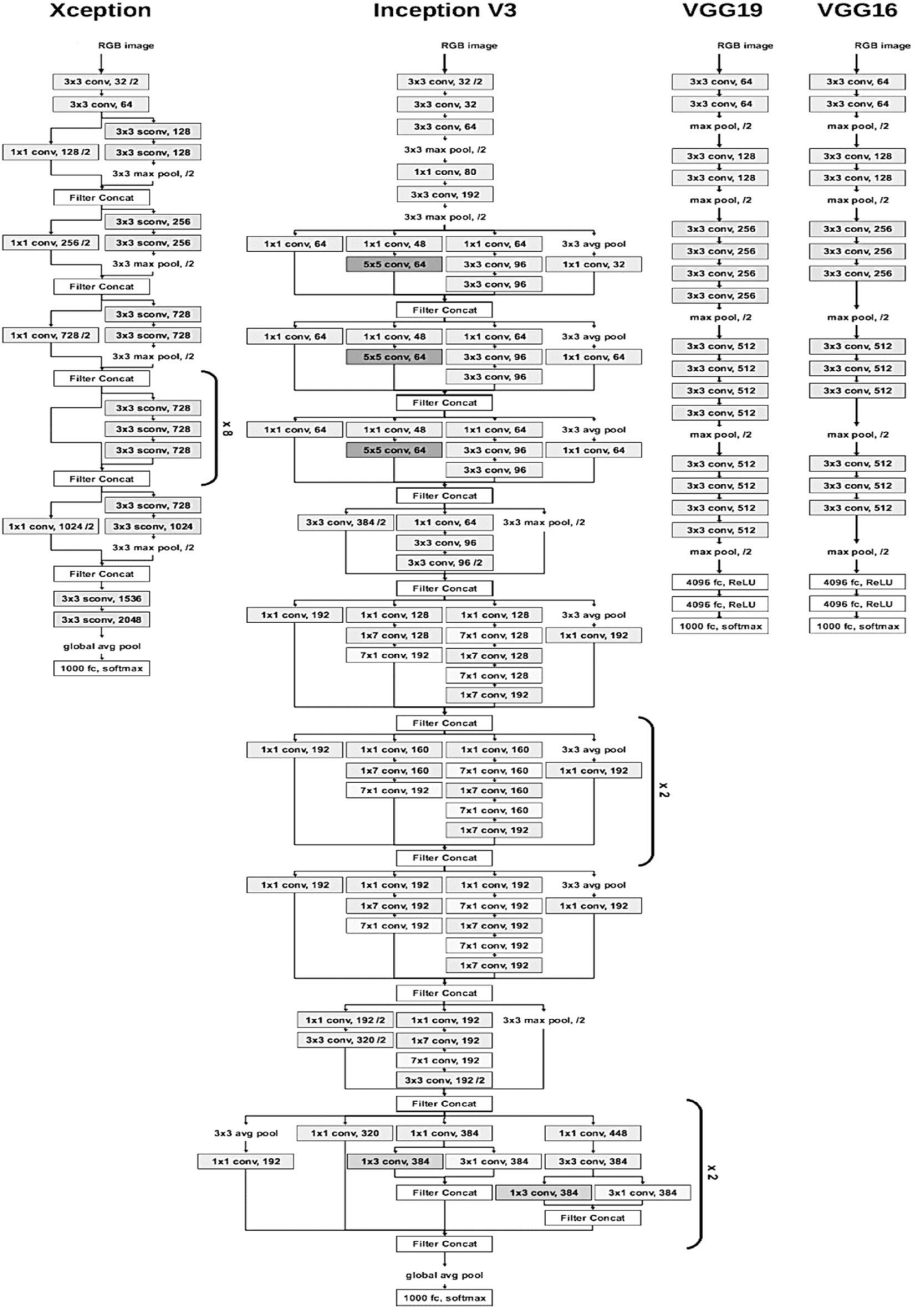
Basic structure of CNNs used in stacking ensemble^39^.

The CNNs used in this stacking ensemble include VGG16, VGG19^36^, Xception^37^, and InceptionV3^38^. These CNNS or so-called base-learners form level 0 of the stacking ensemble. The basic architecture of these base-learners are depicted in Fig. 4. Here, the image size for the CNNs input is 150 × 150 × 3 and the average pooling is used. To use these networks, the last layer is removed and then, flatten, batch normalization, dense, drop-out and dense layers are added to the end one after the other. The added drop-out layer has the factor of 0.35, and the last added dense layer includes 4 units with Softmax activation function to deal with our 4-class classification problem. The Relu activation function is considered for the first added dense layer, while the number of its units is determined using Keras tuner in validation process. Afterwards, by freezing the previous layers, only the added layers are trained with the data in both train and validation folders for 5 epochs. In this step, the Adam optimizer with the learning rate of 0.0001 is selected for training the newly-added layers in each CNN. Now, the added layers have the initial weights.

After validating each CNN, each network is trained for 100 epochs using whole train and validation data. In this second round of the training process, some of the last layers are trained and the previous layers remain frozen, therefore their weights are not adjusted.

The Stochastic Gradient Descent with Momentum (SGDM) is considered as the optimizer. The learning rate, momentum values, and the number of trainable layers for CNNs are listed in Supplementary Table S1 online. Moreover, the categorical cross-entropy is used as loss function which should be minimized in both training processes.

In the training process, two callbacks are used for early-stopping and saving the model with the highest efficiency. Therefore, if there is no improvement in the performance of the model (minimizing the loss function) for a certain number of epochs (patience parameter), the training process is stopped before the maximum number of epochs is met. In this work, the patience parameter equals 25 and the minimization of the loss function is monitored for early-stopping. Also, since the model obtained from completing the training epochs or stopping early (the last trained model) might not necessarily be the best model, saving the model with the highest accuracy and loading it will resolve this issue.

#### Level 1 of stacking ensemble

After training all the base-learners by the training dataset, a meta-learner is presented as level 1 part of stacking ensemble and trained to achieve higher accuracy through combining the results of the trained base-learners. In this study, MLP classifier is used as meta-learner for level 1 of stacking ensemble. This meta-learner is trained on the whole train and validation dataset taking the outputs (predictions) of the base-learners trained in the previous step as input. For this purpose, the predictions from base-learners are stacked and reshaped to be used as input tensors for the MLP model. In fact, the base-learners of the previous step were trained directly by the training dataset, and the MLP model is indirectly trained by the training dataset. To apply obtained model to the new test images, test B-scans should be divided into strips and the resulted strips should be fed to the stacking ensemble model to be classified into one of the mentioned classes. MLP classifier includes 100 hidden layers and maximum iteration parameter is set to be 300.

## Results

In this work, to have a complete evaluation of the proposed classification model, a number of criteria have been considered, which are mentioned in the caption of Table 3.

**Table 3:** Per class and weighted average measures for method in^29^, stand-alone CNNs and stacking ensemble of our method.

Model per class parameter	Inception V3	VGG16	VGG19	Xception	Method in^29^	MLP ensemble
**Accuracy**						
Abnormal	0.948	0.792	0.922	0.883	0.931	0.974
MA	0.883	0.883	0.987	0.922	0.962	0.987
Normal	0.935	0.948	0.922	0.857	0.943	0.961
Vessel	0.896	0.961	0.987	0.974	0.946	1
**Weighted average accuracy**	0.913	0.91	0.958	0.916	0.945	0.982
**Precision**						
Abnormal	0.813	0.464	1	0.619	0.952	0.928
MA	0.682	1	0.945	0.739	0.964	0.944
Normal	0.895	0.944	0.797	1	0.97	0.947
Vessel	0.95	1	0.963	1	0.964	1
**Weighted average precision**	0.851	0.888	0.921	0.873	0.963	0.961
**Recall (sensitivity)**						
Abnormal	0.929	0.929	0.571	0.929	0.86	0.928
MA	0.882	0.471	1	1	0.913	1
Normal	0.85	0.85	0.95	0.45	0.907	0.9
Vessel	0.731	0.885	1	0.923	0.883	1
**Weighted average recall**	0.831	0.792	0.909	0.818	0.890	0.961
**Specificity**						
Abnormal	0.952	0.762	1	0.873	0.866	0.984
MA	0.883	1	0.983	0.9	0.941	0.983
Normal	0.964	0.982	0.912	1	0.86	0.982
Vessel	0.980	1	0.980	1	0.887	1
**Weighted average specificity**	0.95	0.952	0.967	0.818	0.888	0.989
**F1-score**						
Abnormal	0.867	0.619	0.727	0.743	–	0.928
MA	0.769	0.64	0.971	0.85	–	0.971
Normal	0.872	0.895	0.863	0.621	–	0.923
Vessel	0.826	0.939	0.981	0.96	–	1
**Weighted average F1-score**	0.833	0.803	0.902	0.808	–	0.961
**Weighted average ROC-AUC score**	0.981	0.97	0.99	0.989	–	0.998

Accuracy = (TP + TN)/(TP + TN + FP + FN), Precision = TP/(TP + FP), Recall(sensitivity) = TP/(TP + FN), Specificity = TN/(TN + FP), F1score = (2 × Recall × Precision)/(Recall + Precision) = (2TP)/(2TP + FP + FN), ROC-AUC score: the closer this criterion is to one, the greater the number of correctly predicted cases. On the other hand, the closer this criterion is to zero, the greater the number of incorrectly predicted cases.

Here, TP (True Positive) for a specific class represents the number of cases that have been correctly classified in that class and TN (True Negative) represents the number of cases that have been correctly classified as not belong to that class. The FP (False Positive) for a specified class also determines the number of items that were incorrectly predicted in that class, and finally the FN (False Negative) indicates the number of items that were incorrectly predicted in other categories than the class under review.

**Figure 5:**
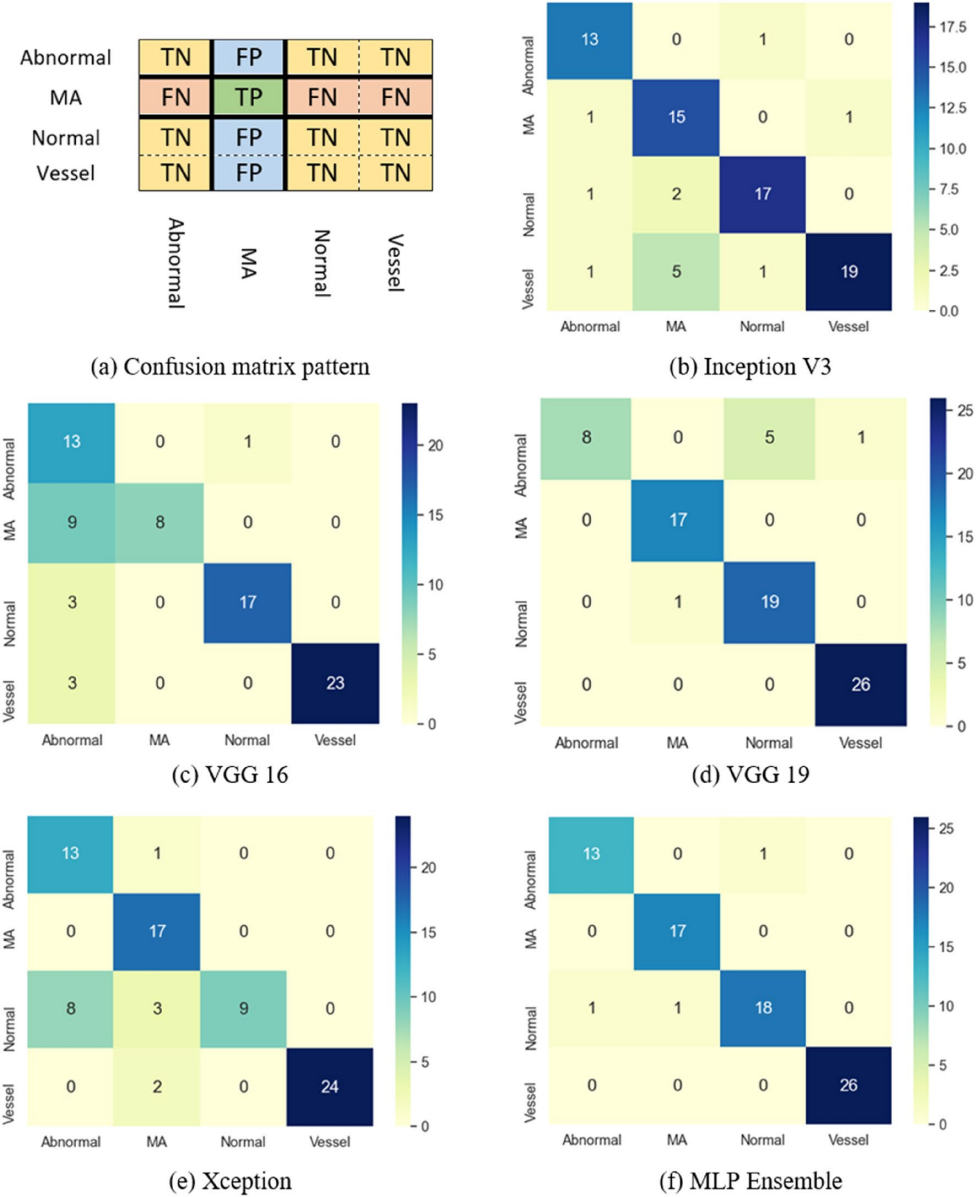
Confusion matrices. (**a**) The relationship between confusion matrix and TP, TN, FP, and FN for MA class. (**b**–**f**) Confusion matrices of different CNNs and stacking ensemble (rows: True labels and cols: Predicted labels) (in color).

As can be seen in Table 3, the above-mentioned measures can be expressed per class for stand-alone CNNs and the stacking ensemble. Also, the measures of different classes can be combined to have a single measure for the model. In the second case, the weighted average is used taking a weighted mean of the measures. The weights for each class are the total number of samples of that class. In addition, Table 3 indicates the weighted average measure for stand-alone CNNs and the stacking ensemble. As can be seen in Table 3, the stacking ensemble has the accuracy of 0.987, 0.961, 0.974, and 1 in classifying MA, normal, abnormal, and vessel strips. Also, from this Table, the overall accuracy of stacking ensemble is 0.982. Some misclassified examples can be found in Supplementary Fig. S4 online. According to Tables 3, proposed method outperforms the method presented in^29^. Figure 5b–f indicates confusion matrices resulted from performing classification utilizing different CNNs and stacking ensemble. The experiment is repeated on the dataset whose test data is prepared from the images of cases who have not been included in the training and validation processes. Confusion matrix is a matrix or table in a way that one axis represents true labels and the other one expresses predicted labels. In this matrix, according to the pair of the true and predicted values for each class label, the matrix entries or the table cell values are calculated. The relationship between confusion matrix and TP, TN, FP, and FN is shown in Fig. 5a for MA class. The results show that the ensemble outperforms each stand-alone CNN. The experiments are repeated on the dataset whose test data is prepared from the images of cases that have not been included in the training and validation processes, and the results are shown in Supplementary Fig. S5 and Supplementary Table 2 online.

## Discussion

In this paper, a method is presented to detect MAs in OCT images using deep convolutional neural networks and transfer learning. For the lack of large labeled datasets which comprise OCT image strips with suitable labels, we have created our dataset. Because MAs and vessels are hard-to-detect objects in OCT images, to create OCT strips with appropriate labels, an accurate image registration method is performed to align OCT images and FA photographs. After that, with the help of corresponding FA images, the OCT strips are created from OCT B-scans in four labels, namely MA, normal, abnormal, and vessel. Once the OCT strips are created and organized as four-class dataset, a stacking ensemble comprising four fine-tuned, pre-trained CNNs is trained to classify the strips of OCT images into the mentioned classes. Once the model is obtained, it can be used to classify the strips from the test B-scans without the need for FA images. The experimental results show that the proposed method classifies OCT image strips and specially detects OCT strips containing MA in a more precise way.

In the current study, we created and organized a dataset containing a limited number of OCT image strips with specific labels, namely MA, normal, abnormal, and vessel. For future works, increasing the number of samples of the dataset enables creating, using, and training deep convolutional neural networks from scratch. Also, analyzing information from the 3D nature of OCT imaging and especially neighboring B-scans can lead to more accurate classification results. For future work, and with a larger dataset, B-scans or OCT volumes can be taught to the CNNs according to their several labels such as normal, MA, etc. without the need for cropping operations, and test data can also be fed as B-scans or volumes to the network.

## Supplementary Information

Supplementary Information.

## Data Availability

The authors declare that the main data supporting the findings of this study are available within the article and its Supporting Information files. Extra data are available from the corresponding author on a reasonable request.
