# Supplementary material for: Automatic detection of microaneurysms in optical coherence tomography images of retina using convolutional neural networks and transfer learning
Source: Sci Rep. 2022 Aug 17;12:13975. doi: 10.1038/s41598-022-18206-8 (PMC9385621; doi:10.1038/s41598-022-18206-8)
Supplement: Supplementary file 1 — Supplementary Information. [file 41598_2022_18206_MOESM1_ESM.docx]

Automatic Detection of Microaneurysms in Optical Coherence Tomography Images of Retina Using Convolutional Neural Networks and Transfer Learning

Ramin Almasi^1^, Abbas Vafaei^1,*^, Elahe Kazeminasab^1^, and Hossein Rabbani^2,*^

1 Department of Computer Engineering, Faculty of Engineering, University of Isfahan, Isfahan, Iran

2 Medical Image & Signal Processing Research Center, Isfahan University of Medical Sciences, Isfahan, Iran

* [rabbani.h@ieee.org](mailto:rabbani.h@ieee.org), [abbas_vafaei@eng.ui.ac.ir](mailto:abbas_vafaei@eng.ui.ac.ir)

| 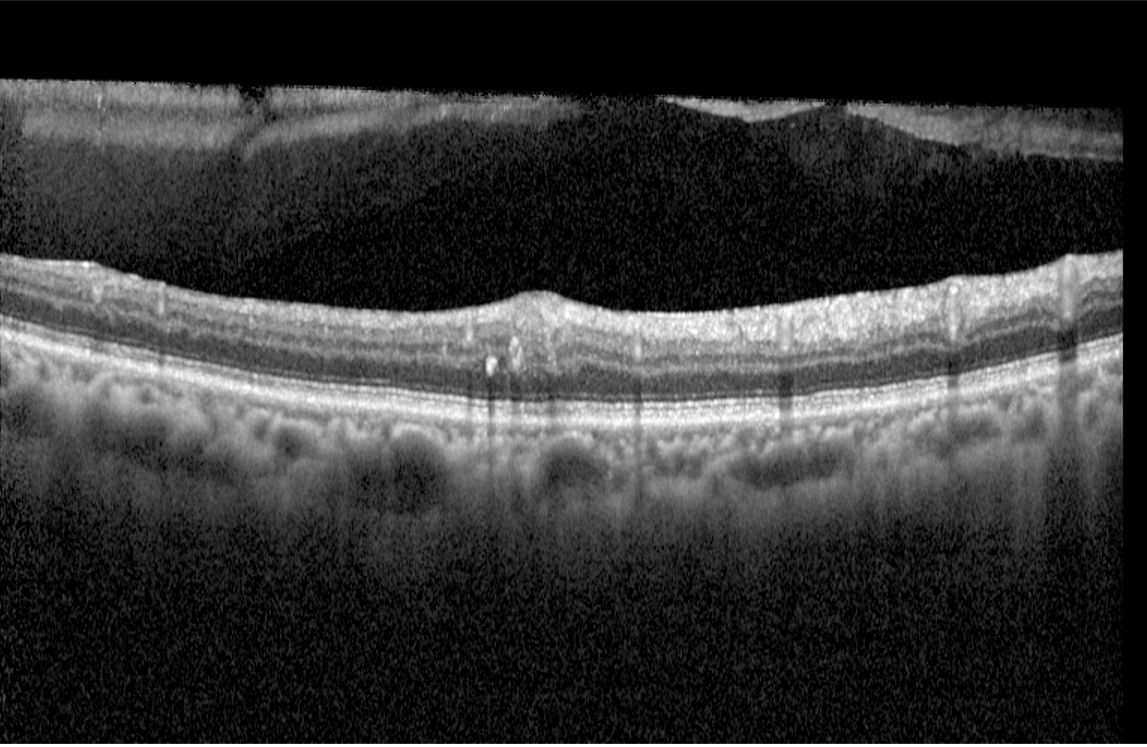 | 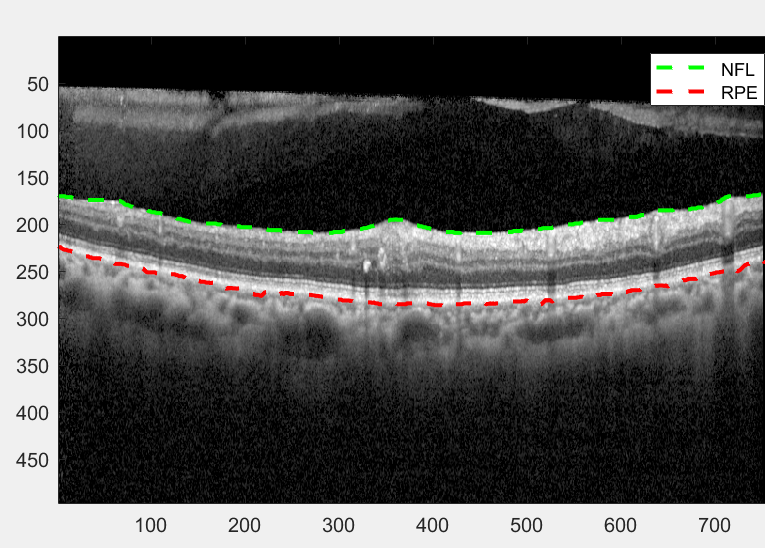 | 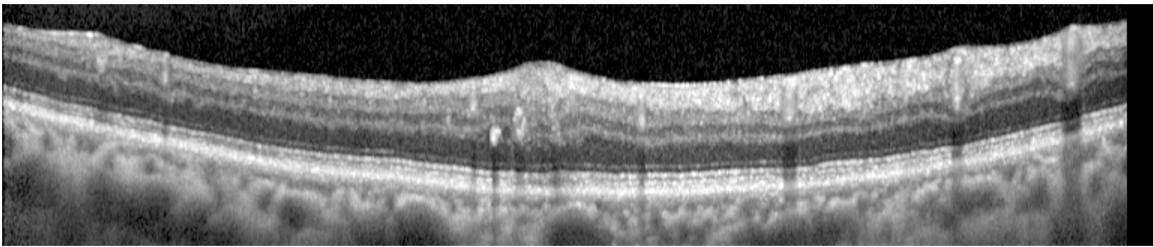 |
| --- | --- | --- |
| (a) | (b) | (c) |

Figure S1. Cropping the OCT image. (a) Original OCT image. (b) Detection of RNFL and RPE layers. (c) Cropping OCT image to include the highest part of NFL and the lowest part of RPE.


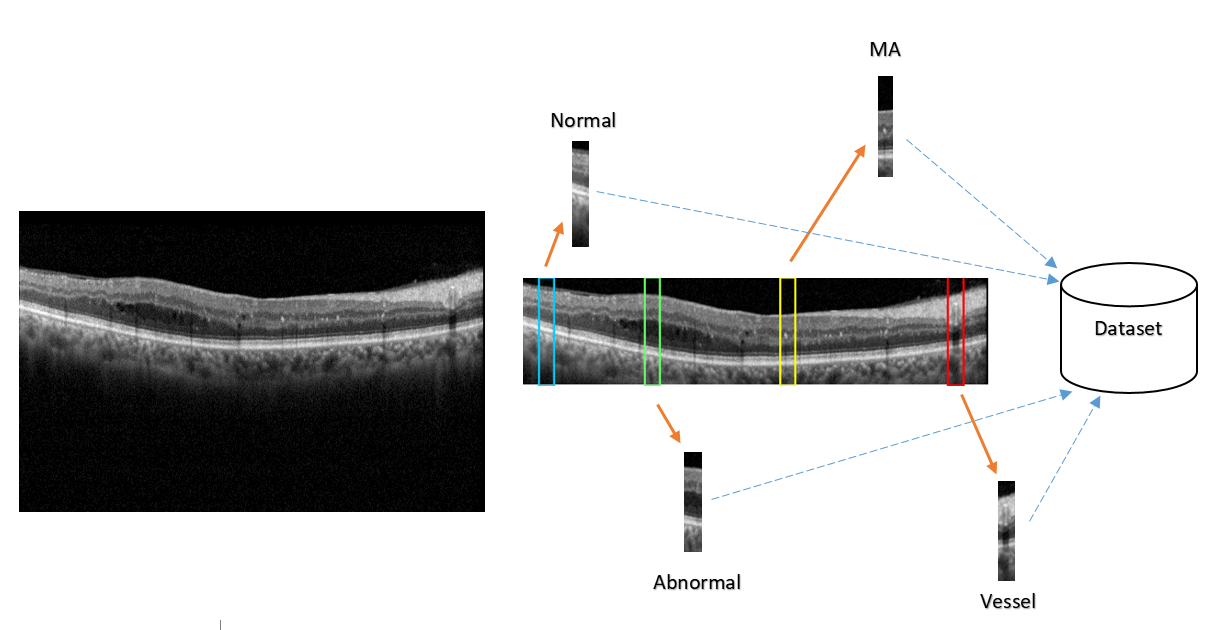


| (a) | (b) |
| --- | --- |

Figure S2. Preparing dataset. (a) An OCT B-scan. (b) Creating dataset of OCT strips from cropped B-scan.


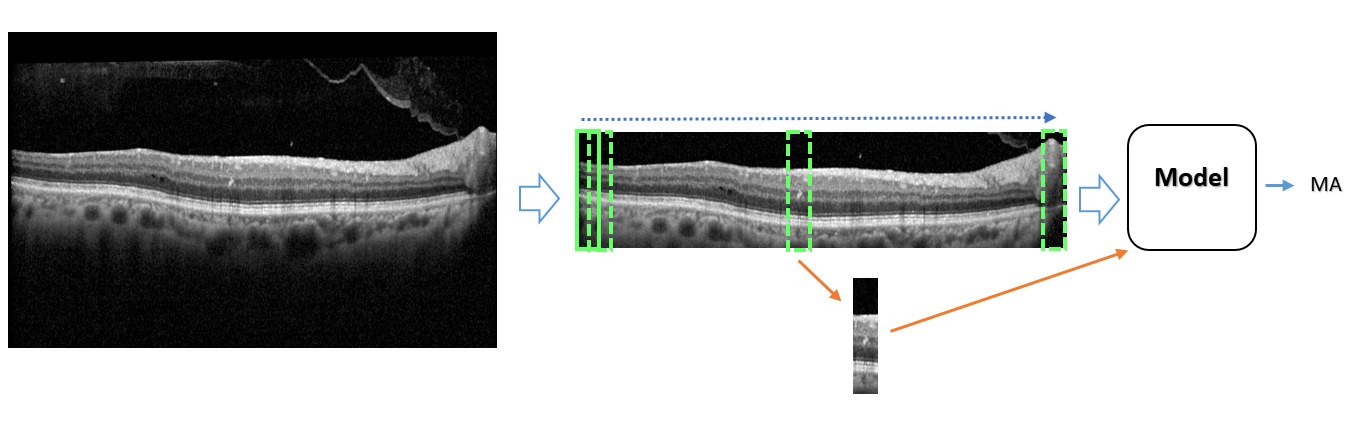


(a)


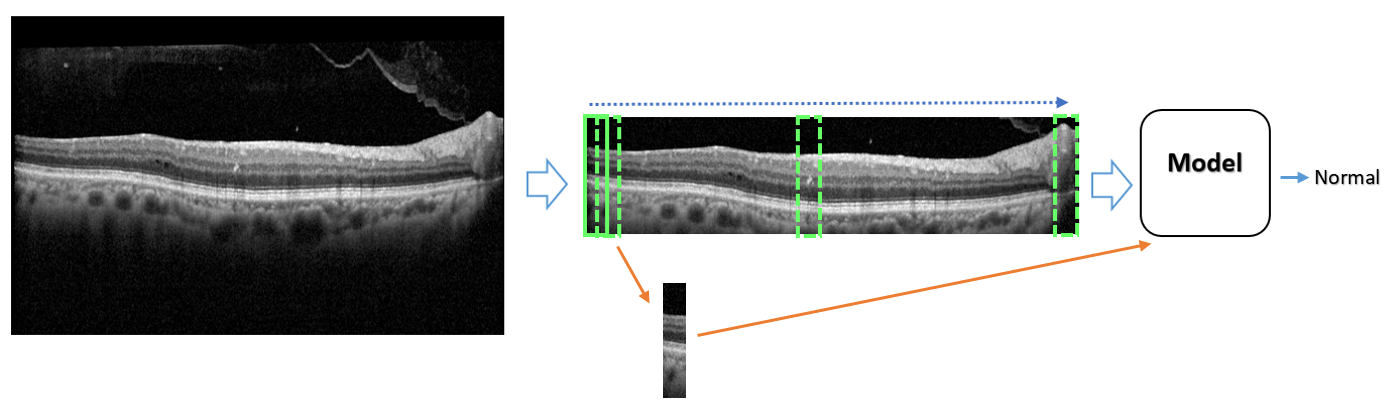


(b)

Figure S3. Testing OCT B-scan strips. (a) Classifying a test OCT strip as MA. (b) Classifying a test OCT strip as normal.

| 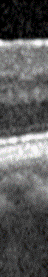 | 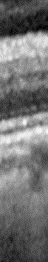 | 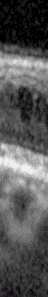 |
| --- | --- | --- |
| (a) | (b) | (c) |

Figure S4. Some misclassified cases. (a) Represents an OCT strip which actually belongs to the normal class, but is incorrectly classified in the MA class. (b) Represents the OCT strip, which is actually in the normal class but is incorrectly categorized in the abnormal class. (c) Represents the OCT strip, which is actually in the abnormal class but is incorrectly categorized in the normal class.

Table S1. The tuned hyperparameters for each CNN

| CNN | Learning rate | Momentum | Dense layer units | Number of trainable layers in base model |
| --- | --- | --- | --- | --- |
| VGG16 | 0.0001 | 0.99 | 64 | 5 |
| VGG19 | 0.00011943 | 0.93574 | 96 | 6 |
| InceptionV3 | 0.0001 | 0.99 | 128 | 42 |
| Xception | 0.0034261 | 0.9 | 128 | 19 |

Table S2. Per class and weighted average measures for stand-alone CNNs and stacking ensemble of our method on the dataset whose test data is prepared from the images of cases who have not been included in the training and validation processes.

| Model  per class  Parameter | | Inception V3 | VGG16 | VGG19 | Xception | MLP Ensemble |
| --- | --- | --- | --- | --- | --- | --- |
| Accuracy | Abnormal | 0.836 | 0.791 | 0.955 | 0.91 | 0.925 |
|  | MA | 0.910 | 0.955 | 0.94 | 0.97 | 0.985 |
|  | Normal | 0.836 | 0.851 | 0.836 | 0.896 | 0.925 |
|  | Vessel | 0.881 | 0.985 | 0.791 | 0.955 | 0.985 |
| Weighted Average  Accuracy | | 0.866 | 0.906 | 0.864 | 0.933 | 0.957 |
| Precision | Abnormal | 0.556 | 0.45 | 0.909 | 1 | 0.889 |
|  | MA | 0.834 | 0.786 | 1 | 0.928 | 1 |
|  | Normal | 0.667 | 0.846 | 0.9 | 0.75 | 0.818 |
|  | Vessel | 0.818 | 0.957 | 0.611 | 0.913 | 0.957 |
| Weighted Average  Precision | | 0.731 | 0.84 | 0.827 | 0.886 | 0.914 |
| Recall  (Sensitivity) | Abnormal | 0.417 | 0.75 | 0.833 | 0.5 | 0.667 |
|  | MA | 0.714 | 0.786 | 0.714 | 0.929 | 0.929 |
|  | Normal | 0.842 | 0.579 | 0.47 | 0.947 | 0.947 |
|  | Vessel | 0.818 | 1 | 1 | 0.954 | 1 |
| Weighted Average  Recall | | 0.731 | 0.791 | 0.761 | 0.866 | 0.910 |
| Specificity | Abnormal | 0.927 | 0.8 | 0.981 | 1 | 0.982 |
|  | MA | 0.962 | 1 | 1 | 0.981 | 1 |
|  | Normal | 0.834 | 0.958 | 0.979 | 0.875 | 0.916 |
|  | Vessel | 0.911 | 0.978 | 0.689 | 0.956 | 0.977 |
| Weighted Average  Specificity | | 0.902 | 0.945 | 0.889 | 0.946 | 0.965 |
| F1-Score | Abnormal | 0.476 | 0.562 | 0.87 | 0.667 | 0.762 |
|  | MA | 0.769 | 0.88 | 0.834 | 0.929 | 0.962 |
|  | Normal | 0.744 | 0.687 | 0.621 | 0.837 | 0.878 |
|  | Vessel | 0.818 | 0.977 | 0.759 | 0.934 | 0.977 |
| Weighted Average  F1-Score | | 0.726 | 0.801 | 0.755 | 0.857 | 0.908 |
| Weighted Average  ROC-AUC Score | | 0.928 | 0.957 | 0.97 | 0.973 | 0.97 |


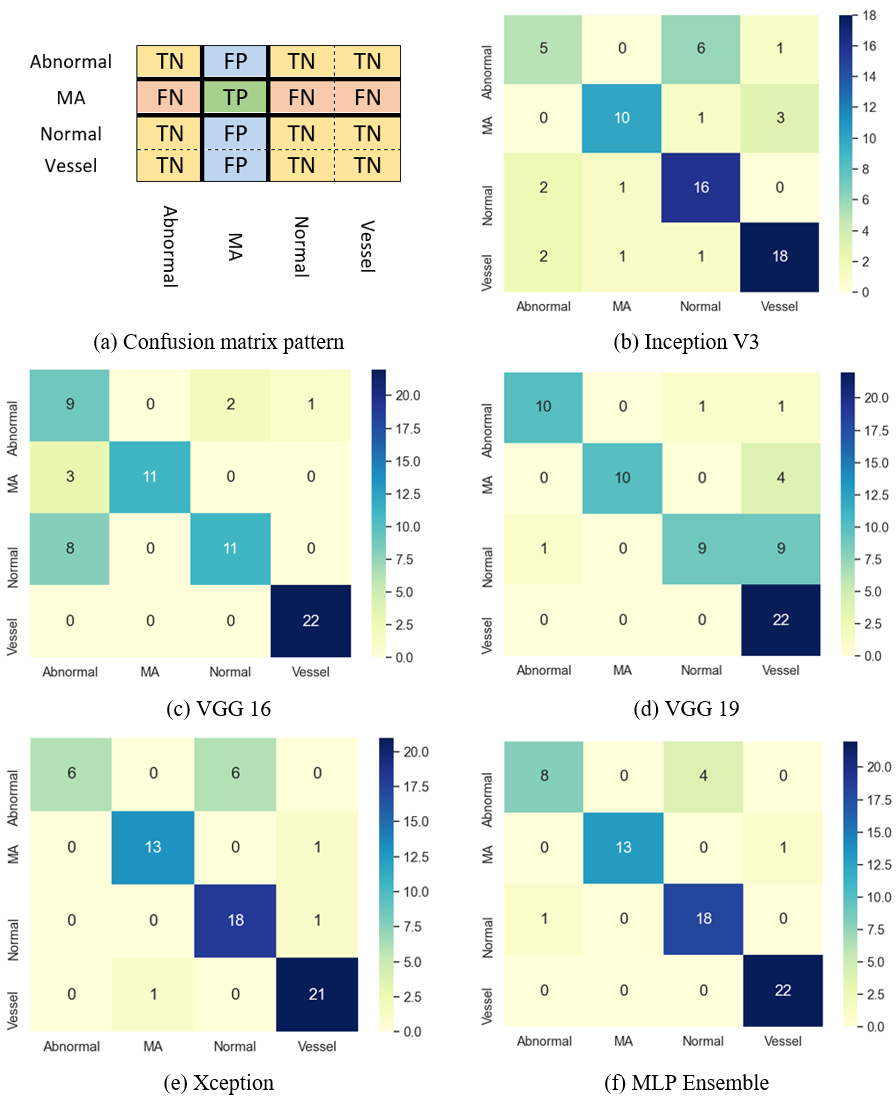


Figure S5. Confusion matrices on the dataset whose test data is prepared from the images of cases who have not been included in the training and validation processes. (a) The relationship between confusion matrix and TP, TN, FP, and FN for MA class. (b-f) Confusion matrices of different CNNs and stacking ensemble (rows: True labels and cols: Predicted labels). (in color)
